# Evaluation of the efficacy of transobturator tape surgery in the treatment of stress urinary incontinence using urodynamics and questionnaires

**DOI:** 10.4274/tjod.46034

**Published:** 2016-12-15

**Authors:** Cihan Aygül, Ramazan Özyurt, Bulat Aytek Şık, Serkan Kumbasar

**Affiliations:** 1 İstanbul Training and Research Hospital, Clinic of Obstetrics and Gynecology, İstanbul, Turkey; 2 İstanbul Aydın University Faculty of Medicine, Department of Obstetrics and Gynecology, İstanbul, Turkey; 3 Sakarya Training and Research Hospital, Clinic of Obstetrics and Gynecology, Sakarya, Turkey

**Keywords:** Stress urinary incontinence, Q-type test, transobturator tape surgery, urodynamic

## Abstract

**Objective:**

To measure the efficiency of transobturator tape (TOT) surgery using urodynamics and questionnaires in stress urinary incontinence.

**Materials and Methods:**

Ninety-two patients with stress and mixed urinary incontinency who underwent TOT surgery were selected for the study. We retrospectively examined the patients’ urodynamics, ultrasonography, demographic characteristics, incontinency surveys, life quality scores [incontinence impact questionnaire, (IQ-7) and urinary distress inventory (UDI-6)], diagnostic findings, Q-type test, surgical records, and complications. Patients treatment adherence, life quality scores, and urodynamics were evaluated as per the findings and complications following discharge of the patients between 12 and 36 months. Patients with a surgical history as the result of incontinence were excluded from the study.

**Results:**

Prior to surgery, 57 (61%, 95) patients had stress urinary incontinence (SUI), and 35 (38%, 05) patients had mixed urinary incontinence (MUI). During surgery, 45 (48%, 91) patients underwent extra pelvic surgical intervention. The mean follow-up time was 22.17±7.55 months. Our subjective success rate was 91%, 3 and the objective success rate was 78%, 3. In the life quality evaluation, a statistically significant improvement was found between IIQ-7 and UDI-6 scores. Parity over 4 was an important failure reason. Two (2%, 17) patients developed vaginal erosion, 2 (2%, 17) of the patients developed temporary urine retention, and 1 (1%, 08) patient developed nova urge incontinence.

**Conclusion:**

Our study demonstrates that TOT surgery provides high objective and subjective success and has a positive impact on life quality. The ease of application and lower complication rate makes TOT a valuable alternative for other treatment approaches in the surgical treatment of SUI.

## INTRODUCTION

The International Continence Association (ICS) described Urinary Incontinence (UI) as unintended urine continence that becomes a social and hygienic problem^(1)^. Two hundred fifty million adult individuals are effected by UI around the world, between 7-37% of women aged between 20-39 years experience UI^(2,3)^. In Turkey, it is known that the prevalence of UI in women ranges between 16.4% and 49.7%^(4)^. Incontinence is a serious issue that results in loneliness, economic problems, and has a negative effect on an individual’s sexual life by causing shame, declining confidence, and a decrease in social activities^(5)^. Although there are many risk factors that result in incontinence, the most apparent risk factors in women are old age and trauma experienced during delivery through pregnancy and higher parity numbers, and births with interventions and tears^(6,7,8,9)^.

The most common type of incontinence experienced in women is stress urinary incontinence (SUI), which is seen commonly in middle aged and parous women, and experienced as a result of situations that increase the pressure on the abdomen such as coughing, laughing, and heavy lifting. Stress incontinence was described by the ICS as UI that occurs as a result of intravesical pressure overrunning urethra pressure without any increase in detrusor activity. Stress incontinence as an incontinence type, is the variety that patients can benefit the most from surgery among the treatment options. Although there are different techniques available in treatment, there is no agreement as to which surgical intervention is the most efficient and appropriate for which patient. We use the classic external to internal transobturator tape (TOT) technique for patients who present to our clinic with SUI or mixed urinary incontinence (MUI). In this study we aimed to compare the subjective and objective success of TOT surgery with other surgical techniques, to demonstrate the short- and long-term complications, and its effects on patients’ quality of life in light of the literature.

## MATERIALS AND METHODS

Our study comprised 125 patients with MUI and SUI who were treated with TOT surgery between April 2010 and April 2012 in İstanbul Training and Research Hospital, Gynecology and Obstetrics clinic. In line with data integrity and patient compliance, 92 of the patients were included in our study. The study was conducted in İstanbul Training and Research Hospital, Perinatology and Delivery Unit, following receipt of approval from the Hospital Training and Planning Committee and Committee of Ethics. Within the indicated dates, patients considered within the scope of the study were informed and included in the study after receiving their signed form of approval. Patients who had urge incontinence, undergone surgery for incontinence, had a significant neurologic disorder, had undergone previous vaginal surgery, or used medication that created a tendency to bleed were excluded from the study. We retrospectively examined the patients urodynamics, ultrasonography, demographic characteristics, incontinence surveys, life quality scores [incontinence impact questionnaire, (IQ-7) and urinary distress inventory (UDI-6)], diagnostic findings, Q-type test, surgical records, and complications. Also, we reviewed the patients’ treatment adherence, life quality scores, urodynamics as evaluated per findings and complications following the discharge of the patients between 12 and 36 months. In the pre-operative period, the histories of the patients were collected and pelvic inspections, urinalysis, [(life quality tests: UDI-6 and incontinence impact questionnaire-7 (IIQ-7)], Q-type tests, urodynamics, and residual urine tests were conducted. The Baden-Walker classification was used to classify pelvic limb prolapsus in the pelvic inspection. Cough stress tests were positive for all the patients in our study. Cervix vesicae mobility was evaluated with Q-type test. If there was no reproduction in the urine culture taken prior to urodynamic testing, patients were proceeded to surgery; if reproduction was observed, patients were re-evaluated following appropriate antibiotic treatment. For conducting multi-channel cystometry, 8 F micro-type transducers (2 transducers positioned on the tip and 6 cm behind, positioned in a 3 o’clock direction) were placed transurethrally in the patient. Pressure measurements were conducted by connecting the system to the patient who was laid on the patient examination couch as they drained their urine, and examined for residual urine. The bladder was filled with sterile physiologic serum at 50 cc/minute at room temperature. During the filling procedure, the time to first sensation of urine, and first urge to urinate, normal urge to urinate, strong urge to urinate, and maximal bladder capacity (maximum pressure level of the patient before urinary incontinence) were evaluated. During measurements following the 200 cc physiologic serum, UI was induced using the Valsalva maneuver in the lithotomy position. It was re-applied as 100 cc if no continence was observed. Valsalva leak point pressure values (VLPP) were considered for defining SUI sub types. Intrinsic sphincter insufficiency was diagnosed for the patients with VLPP below or equal to 60 cm H_2_O, and type 2 SUI was diagnosed for the patients with VLPP above or equal to 90 cm H_2_O. Detrusor dysfunction was diagnosed using urodynamics through an immediate increase in basal detrusor pressure as 15 cm H_2_O or more during the filling procedure.

For the decision of surgery, patient symptoms and urodynamic parameters were considered together. TOT procedures were conducted as external to internal as described by Delorme. All operations were performed using outside-in Obtryx (Boston Scientific, Natick, MA, USA) brand kits. All patients were administered intravenous 2 gr cefazolin sodium prior to surgery. A longitudinal incision of about 2 cm was made starting 0.5 cm from under the urethral meatus to the front wall of the vagina. Periurethral dissection was performed with blunt and sharp dissection until below the ischiopubic ramus. A bilateral 5-mm incision was made at the clitoris level, the lateral side of the labium majus, 15 mm lateral of the ischiopubic ramus. A hook-shaped TOT needle was advanced medially by palpating the posterior of the ischiopubic ramus and m. obturatorius internus with an index finger, which was on the paraurethral dissection point, and passed through the dermis, obturator membrane, and incision in the vagina, respectively. After this procedure, we checked whether vaginal fornix and urethra perforation had occurred. The mesh-attached needle was removed from the dermis from the reverse side. The same method was applied to the other side. The strain of the mesh was adjusted to leave an opening to allow for a scissor tip to enter between the urethra and band at the end of the procedure. At the end of the procedure, an 18 F foley catheter was placed. The duration of TOT and other additional surgical procedures (if applicable) was recorded.

Additional pelvic floor interventions were performed on patients according to the indications. The patients’ catheter were removed on postoperative day 1 and we waited for spontaneous urination. As a result of the evaluations, 57 patients with SUI and 35 patients with MUI were included in the study.

### Statistical Analysis

SPSS version 11.0 was used for the statistical analysis of this study (Statistical Package for the Social Sciences Inc; Chicago, IL, USA). Student’s t-test, the Mann-Whitney U, paired t-test, Wilcoxon rank test, Fisher’s exact test, and chi-square tests were used for comparisons. P<0.05 were accepted as statistically significant.

## RESULTS

A total of patients 125 underwent TOT. In line with data integrity and patient compliance, 92 patients were included in our study. The average of the patients was 48.46 years (range, 29- 83 years). Fifty patients were premenopausal and 42 were postmenopausal patients. None of the postmenopausal patients were receiving hormone replacement treatment. The average menopause duration for patients who were postmenopausal was 10.12 years (range, 1-40 years). The mean follow-up duration was 22.17 months (range, 12-36 months). The average parity was 3.42 (range, 1-15); 4 patients had a history of cesarean section, and 2 patients had a history of vacuum-assisted birth. The average baby birth weight was 3708.26 gram (range, 2600-6000 g). The average duration of incontinence was 6.83 years (range, 1-40 years). The average body mass index (BMI) was calculated as 28.51± 4.3 kg/m^2^ (range, 23.3-39.5 kg/m^2^). According to BMI, 58.69% of the patients were overweight and 33.69% were obese. No patients were morbidly obese. Forty-one patients had systematic diseases: hypertension (n=16), diabetes (n=8), thyroid disease (n=5), chronic obstructive pulmonary disease (COPD) (n=6), hypertension + diabetes (n=4), and hypertension + COPD (n=2). Twenty (21.73%) patients had undergone previous gynecologic operations, none of which were in relation with incontinence; 6 (6%, 52) operations were hysterectomy. Thirty-five patients (38.05%) were diagnosed as having MUI and 57 patients (61.95%) were diagnosed as having SUI as a result of anamnesis, examinations, and urodynamic inspections. Patients with pure urge incontinence were excluded. All patients had symptoms of stress incontinence and cough stress test results were positive. Table 1 shows the demographic and clinical characteristics of the patients (Table 1).

All patients underwent external to internal TOT procedures. Forty-five (48.91%) patients underwent additional procedures as per indication during the same surgical period: vaginal hysterectomy + anterior colporrhaphy (VAH + CAP) (n=5), anterior colporrhaphy (n=3), posterior colporrhaphy (n=23), manchester (n=3), anterior posterior colporrhaphy (n=4), abdominal hysterectomy + bilateral salpingo-ophorectomy (total abdominal hysterectomy + buthionine sulfoximine) (n=4), bilateral tube ligation, cyst extirpation (n=1).

No intraoperative complications developed in the patients in the present study. No hematomas, wound site infections, or urinary system infections developed during the postoperative stage. Two (2.17%) patients developed a foreign body reaction. However, no interventions were conducted on the patients because the erosion in the vagina was less than 1 cm and asymptomatic. The patients were taken to follow-up. One (1.08%) patient developed incontinence and this patient was treated with anticholinergic agents.

The 35 patients with MUI were started on trospium chloride one week prior to operation and treatment continued following the operation. Patients were re-evaluated after an average of 22.17 months (range, 12-36 months). Patients were primarily evaluated at follow-up examinations as per their symptoms. Examinations, urodynamics, Q-type test, UDI-6, and IIQ surveys were conducted: one patient (1.1%) had worsened symptoms and 12 (13%) patients’ symptoms were improved, no significant changes were observed in 7 (7.6%) patients, and 72 (78.3%) were symptom free. The results were compared with the results obtained at the preoperative stage.

The first two questions of the UDI-6 survey are regarding symptoms of irritation and the 4^th^ question is about stress symptoms, the 5^th^ and 6^th^ questions are related with obstruction; these questions are evaluated by grouping them individually. An average 1.02 score decrease was seen in the pre- and post-operative UDI-6 evaluations for the 1^st^ and 2^nd^ questions, an average 3.66 score decrease was seen for the 4^th^ question, and a 4.57 score decrease was found for the 5^th^ and 6^th^ questions. All questions in UDI-6 and IIQ-7 saw an average score decrease of 10.86. The decrease in each evaluation was found statistically significant, which proved the positive effect on life quality (p<0.01). The 5^th^ and 6^th^ questions of UDI-6 had no statistically significant change (p>0.05) (Table 2).

Seventy-two the patients (78.3%) were identified as having objective cure when the post-operative urodynamics were evaluated. Stress incontinence remained in 20 (21.7%) patients. Objective success was obtained in 65 out of 81 (80.2%) patients with anatomic incontinence, and 7 out of 11 (63.6%) patients with type 3 incontinence. There were no statistically significant differences between TOT success and stress anticontinence subtypes.

Regarding the patients with successful and unsuccessful operations, it was found that parity over 4 was an important reason for failure. Age, BMI, heavy baby birth weight, and duration of symptoms had no significance over the success of the operation (Table 3).

Forty-five (48.91%) patients underwent additional operations as per the indication during the same operative period. There was no significant difference regarding TOT success between patients with without additional pelvic surgical operations. Six (6.5%) patients had a history of hysterectomy; no statistically significant difference was observed between patients with and without a history of hysterectomy.

There was no statistically significant difference in the time to first sensation of urine and maximum bladder capacity regarding the pre-operative and post-operative cystometry values. There was a significant decline in the frequency of daytime and nighttime micturition among the patients (p<0.01). There were no significant difference between the residual urine quantity after an average of 22.17 months for the pre- and post-operative periods (p>0.05). According to the Q-type test results, there was no significant change in the mobility of the cervix vesicae (p>0.05) (Table 4).

## DISCUSSION

The TOT procedure is commonly used in the treatment of UI. One of the most important characteristics that distinguishes TOT procedures from other sling operations is the low rate of complications. The most important complications of the burch procedure are difficulties with urination and an increase in prolapsus after the operation^(10)^. For transvaginal tape procedures, complications include major limb injuries, bladder perforation, and bleeding^(11)^. Although the low rate of complications were remarkable when the results of the TOT procedures were first published, an increase in general complication rates and different complications indigenous to TOT were reported as the follow-up durations and number of cases increased. Along with the possibility of experiencing extensive bleeding, wound site infections, obturatory abscess, urinary retention, and re-operation related with mesh erosion, leg and groin pains were also reported for TOT operations^(12)^. Madjar et al.^(13)^ suggested considering abdomen and pelvis tomography for patients with abdomen pain or urinary symptoms following sling procedures. The risk of vaginal erosion is between 0% and 2.7% following the TOT procedures^(14)^. The most important complication observed in our study was vaginal erosion (n=2, 2.17%).

Post-operative urinary retention can be connected to edema or pain but dysuria or urine retention that continues after one week must be considered seriously. If necessary, the tape must be loosened with traction and re-inserted properly using the same vaginal incision^(15)^. In another study, bladder exit obstruction was identified in 3.8% of participants following TOT, and it was stated that removal or loosening of the tape as early as possible could be beneficial in the presence of a clinically significant obstruction^(16)^. In our study 2 patients (2.17%) had temporary urine retention following the operation. For these patients catheters were left in the bladder for 1 week. Patients urinated comfortably after the removal of the catheters and no additional operations were required because the residual urine quantity was below 100 mL. One patient (1.08%) had novo urge incontinence in the post-operative period; this rate was 2% in the study conducted by Juma and Brito^(17)^.

For sling operations, it is not necessary to fix the urethral mobility. On the contrary, continuation of urethral mobility in the post-operative period provides dynamic bending for the urethra during stress^(18)^. In our study, urethral mobility was observed as continuous in the Q-type test, and there were statistically significant changes in the pre- and post-operative values.

In our study, UDI-6 and IIQ-7 forms, which were developed by Uebersax, were used to evaluate life quality before and after the TOT procedures. These forms have been used to evaluate life quality in most studies related with TOT^(17,19)^. The patients’ survey answers were grouped, scored, and the pre- and post-operative scores were compared. When the UDI-6 stress (3^rd^ and 4^th^ questions) and urge (1^st^ and 2^nd^ questions) values were evaluated, the scores were found significantly decreased. No changes were noted in the obstructive (5^th^ and 6^th^ questions) scores. Similar results were reported in the studies conducted by Juma and Brito^(17)^ and Grise et al.^(20)^. The decline of both stress and urge symptoms reminded us of the integral theory of Petros and Ulmsten^(21)^. Our data are in accordance with this theory.

There was a statistically significant increase in patients’ life quality scores in the post-operative period in IIQ-7 scores, which shows that the operations were successful. During the follow-up evaluations as per the symptoms, 12 (13.04%) patients reported a decline in incontinence symptoms, and 72 (78.03%) patients were considered cured. According to this result, 91.3% of patients achieved subjective success and this result is in accordance with the literature. Subjective success rates range between 78% and 91% in the literature^(19,20)^. Seven patients (7.06%) reported no significant change and 1 (1.08%) patient described worsened symptoms and with urge incontinence being added to their stress incontinence. According to the general urodynamic results, 72 (78.3%) patients obtained objective cure. Objective success rates in the literature range between 75% and 89.3%^(20,22)^.

The number of pregnancies and parity, UI level, unsuccessful surgical operations in the past, VLPP ≤60 cm H_2_0, and BMI <30 the operation observed in the study conducted by Rodriguez et al.^(23)^ as not effecting the unwanted side effects and surgical success. In the same study, the effects of the urodynamic measures on SUI surgical treatment cure rates and VLPP were found not to affect the surgical treatment result. In our study, no significant effect for age, duration of symptoms, type of incontinence, heavy baby birth weight and BMI was found to affect treatment success. Parity over 4 was identified as a significant reason for failure.

TOT is commonly conducted with other interventions. In a study that investigated whether the effects of the procedures on the pelvic floor affected TOT success rates, only 2 patient groups were compared; those who underwent TOT procedures, and patients with additional operations^(24)^. In that study, the authors found that additional pelvic interventions had no effect on TOT success. Forty-five patients (50.6%) underwent additional pelvic operations besides TOT. No significant difference was found between patients with and without additional operations in terms of TOT operation success.

## CONCLUSION

Stress incontinence continues to be an important health issue. TOT is a method that is easy to apply with low complication rates and a high rate of success. Our results show the high success rate and low complication rate of TOT procedures. In our study, TOT was considered an efficient and reliable method in accordance with the success rates obtained. However, there is a need for further random prospective studies with different methods and large populations in which long-term results are reported and compared.

## Figures and Tables

**Table 1:**
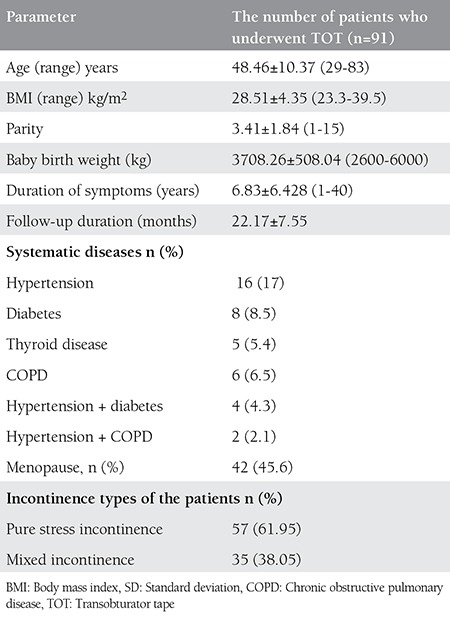
Demographic and clinical characteristics of the patients

**Table 2:**
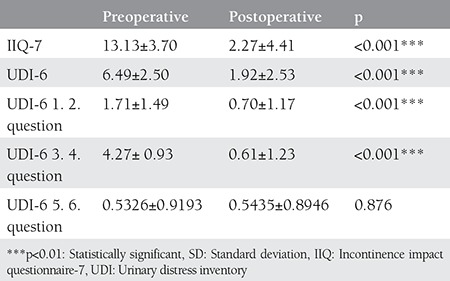
Evaluation of urinary distress inventory-6 and incontinence impact questionnaire-7 scores of the patients

**Table 3:**
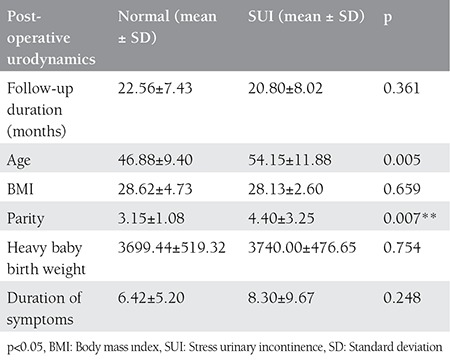
Effects of various parameters on objective success

**Table 4:**
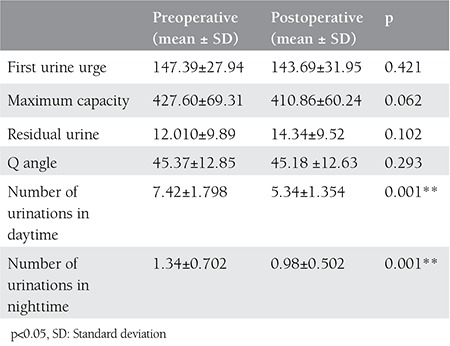
Changes in cystometry values

## References

[1] Abrams P, Andersson KE, Birder L, Brubaker L, Cardozo L, Chapple C, et al (2010). Fourth International Consultation on Incontinence Recommendations of the International Scientific Committee: Evaluation and treatment of urinary incontinence, pelvic organ prolapse, and fecal incontinence. Neurourol Urodyn.

[2] Beji NK, Ozbas A, Aslan E, Bilgic D, Erkan HA (2010). Overview of the social impact of urinary incontinence with a focus on Turkish women. Urol Nurs.

[3] Buckley BS, Lapitan MC, Epidemiology Committee of the Fourth International Consultation on Incontinence P (2010). Prevalence of urinary incontinence in men, women, and children--current evidence: findings of the Fourth International Consultation on Incontinence. Urology.

[4] Basak T, Kok G, Guvenc G (2013). Prevalence, risk factors and quality of life in Turkish women with urinary incontinence: a synthesis of the literature. Int Nurs Rev.

[5] Bilgili N, Akın B, Ege E, Ayaz S (2008). Kadınlarda üriner inkontinans sıklığı ve etkileyen risk faktörleri. Turkiye Klinikleri J Med Sci.

[6] Onur R, Deveci SE, Rahman S, Sevindik F, Acik Y (2009). Prevalence and risk factors of female urinary incontinence in eastern Turkey. Int J Urol.

[7] Zhu L, Li L, Lang J, Xu T, Wong F (2010). Epidemiology of mixed urinary incontinence in China. Int J Gynaecol Obstet.

[8] Işıklı B, Yenilmez A, Kalyoncu C (2011). Eskişehir Alpu ilçesi 18 yaş üstü kadınlarda üriner inkontinans, risk faktörleri ve yaşam kalitesine etkisi: bir toplum tabanlı çalışma. Nobel Med.

[9] Kirss F, Lang K, Toompere K, Veerus P (2013). Prevalence and risk factors of urinary incontinence among Estonian postmenopausal women. Springerplus.

[10] Kjolhede P (2005). Long-term efficacy of Burch colposuspension: a 14-year follow-up study. Acta Obstet Gynecol Scand.

[11] Latthe PM, Foon R, Toozs-Hobson P (2007). Transobturator and retropubic tape procedures in stress urinary incontinence: a systematic review and meta-analysis of effectiveness and complications. BJOG.

[12] Dursun P, Bildaci TB, Zeyneloglu HB, Kuscu E, Ayhan A (2011). Transobturator tape operation is more effective in premenopausal women than in postmenopausal women with stress incontinence. Korean J Urol.

[13] Madjar S, Frischer Z, Nieder AM, Waltzer WC (2006). Bladder wall abscess following midurethral sling procedure. Int Urogynecol J Pelvic Floor Dysfunct.

[14] Krauth JS, Rasoamiaramanana H, Bartela H BP, Grisard-Anafd M, Lienharte J, et al (2005). Suburethral tape treatment of female urinary incontinence - morbidity assessement of the trans-obturator route and a new tape (I-STOP): a multi-centre experiment involving 602 cases. Eur Urol.

[15] Riva D, Sacca V, Tonta A, Casolati E, Luerti M, Banfet G (2006). TVT versus TOT: a randomized study at 1 year follow up. Int Urogynecol J Pelvic Flor Dysfunct.

[16] Deval B, Ferchaux J, Berry R, Gambino S, Ciofu C, Rafii A, et al (2006). Objective and subjective cure rates after trans-obturator tape (OBTAPE) treatment of female urinary incontinence. Eur Urol.

[17] Juma S, Brito CG (2007). Transobturator tape (TOT): Two years follow-up. Neurourol Urodyn.

[18] Lo TS, Wang AC, Horng SG, Liang CC, Soong YK (2001). Ultrasonographic and urodynamic evaluation after tension free vagina tape procedure (TVT). Acta Obstet Gynecol Scand.

[19] Porena M, Kocjancic E, Costantini E, Giannantonia A, Ranzonic S, Mearinia L, et al (2005). Tension free vaginal tape vs transobturator tape as surgery for stress urinary incontinence: results of a multicentre randomised trial. . Neurourol Urodyn.

[20] Grise P, Droupy S, Saussine C, Ballanger P, Monneins F, Hermieu JF, et al (2006). Transobturator tape sling for female stress incontinence with polypropylene tape and outside-in procedure: prospective study with 1 year of minimal follow-up and review of transobturator tape sling. Urology.

[21] Petros PE, Ulmsten UI (1990). An integral theory of female urinary incontinence. Experimental and clinical considerations. Acta Obstet Gynecol Scand Suppl.

[22] Houwert RM, Venema PL, Aquarius AE, Bruinse HW, Roovers JP, Vervest HA (2009). Risk factors for failure of retropubic and transobturator midurethral slings. Am J Obstet Gynecol.

[23] Rodriguez LV, Almeida F, Dorey F, Raz S (2004). Does Valsalva leak point pressure predict outcome after the distal urethral polypropylene sling? Role of urodynamics in the sling era. J Urol.

[24] Mellier G, Mistrangelo E, Gery L, Philippe C, Patrice M (2007). Tension-free obturator tape (Monarc Subfascial Hammock) in patients with or without associated procedures. Int Urogynecol J Pelvic Floor Dysfunct.

